# Supplementary material for: The genome sequence of the Dark-triangle Button moth, Acleris laterana (Fabricius, 1794)
Source: Wellcome Open Res. 2025 Apr 30;10:231. [Version 1] doi: 10.12688/wellcomeopenres.24003.1 (PMC12449689; doi:10.12688/wellcomeopenres.24003.1)
Supplement: Peer review history

# Reviewer report: Reviewer response for version 1

**DOI:** 10.21956/wellcomeopenres.26481.r130159

**Published:** 2025-09-27

**Authors:** Annabel Whibley

**Affiliations:** 1 Grapevine Improvement, Bragato Research Institute, Lincoln, New Zealand; 2 The University of Auckland, Auckland, New Zealand

Hutchinson and colleagues report the reference genome sequence of the Dark-triangle Button moth, *Acleris laterana.  *A high-quality reference genome assembly (formally 5.C.Q58) is reported here, along with extensive evaluation data and an automatic gene annotation (with no RNAseq input from this specimen).  The reporting of methods, which includes barcode verification of specimen identity, ultra-low input to PacBio sequencing and HiC library preparation. The Data note follows DToL templates, with appropriate methods, and comprehensive descriptions of these and associated metadata. Public accession links are active.

 I wondered if the use of different individuals for the PacBio and HiC datasets might explain some of the failure to scaffold the residual unplaced contigs. Although the total number of scaffolds is relatively large (n>500), this breaks down into  the 30 chromosomal scaffolds and also a large number of considerably smaller scaffolds that collectively contribute < 2% of the total assembly length.  The Z chromosome of this assembly is also striking- at >87Mb it comprises ~15% of total assembly length and falls well outside the range observed for the autosomes (~9-21Mb).

Are sufficient details of methods and materials provided to allow replication by others?

Yes

Is the rationale for creating the dataset(s) clearly described?

Yes

Are the datasets clearly presented in a useable and accessible format?

Yes

Are the protocols appropriate and is the work technically sound?

Yes

Reviewer Expertise:

Bioinformatics, Genomics

I confirm that I have read this submission and believe that I have an appropriate level of expertise to confirm that it is of an acceptable scientific standard.

# Reviewer report: Reviewer response for version 1

**DOI:** 10.21956/wellcomeopenres.26481.r130164

**Published:** 2025-09-19

**Authors:** Theo Leger, Josh Penalba

**Affiliations:** 1 Leibniz-Institut für Evolutions- und Biodiversitaetsforschung, Berlin, Germany; 2 Museum für Naturkunde Berlin, Leibniz-Institut für Evolutions- und Biodiversitaetsforschung Invalidenstr, Berlin, Germany

The authors present the first chromosome-level genome of *Acleris laterana*. Identification of the specimen was checked both morphologically and molecularly with DNA barcoding following published protocol. The sequencing technology (PacBio) and downstream analyses (denovo assembly, assembly curation and quality assessment) are adapted to reconstruct high-quality genomes. Following points should be clarified:

 (1) for PacBio HiFi sequencing, it says “samples were normalized to 20 ng of DNA”. Since only ilAclLate2 was used for PacBio, does that mean only one sample of 20 ng was used or the extraction was divided into multiple subsamples of 20 ng? If so, how many?

 (2) How many SMRT cells were used for the PacBio HiFi sequencing?

 (3) How much sequencing was done on the Illumina NovaSeq6000 in terms of lanes or flow cells?

 We would opt for minor revision.

 The manuscript can be accepted for indexing after completing the information above.

Are sufficient details of methods and materials provided to allow replication by others?

Yes

Is the rationale for creating the dataset(s) clearly described?

Yes

Are the datasets clearly presented in a useable and accessible format?

Yes

Are the protocols appropriate and is the work technically sound?

Yes

Reviewer Expertise:

systematics, phylogenomics

We confirm that we have read this submission and believe that we have an appropriate level of expertise to confirm that it is of an acceptable scientific standard.

# Reviewer report: Reviewer response for version 1

**DOI:** 10.21956/wellcomeopenres.26481.r130161

**Published:** 2025-09-12

**Authors:** Panagiotis Ioannidis

**Affiliations:** 1 Foundation for Research & Technology - Hellas, Crete, Greece

This paper describes the sequencing and assembly of the lepidopteran Acleris laterana. The methodology is the one routinely used by all other DToL projects, which resulted in a high quality assembly, suitable for downstream analyses.

 I have two comments that I would like the authors to address:

 1) In the blobplot (figure 3) there is a "cloud" of scaffolds with less than expected sequencing coverage, even though their GC content is the expected. What is the (putative) origin of these fragments? Are they from A. laterana? Are they bacterial sequences? Are they contaminants from other sources (e.g. food)?

 2) The BUSCO scores seem a bit low compared to that from other sequencing projects. More specifically, the authors mention that there are 94.4% complete BUSCOs, whereas in most other genome assemblies I've recently reviewed this is close to 99%. Do the authors have an explanation why the completeness of this assembly is lower than usual? And also could this be linked to the previous observation on the blobplots? What I mean is could it be that some of the fragments with lower coverage are legit A. laterana sequences that should be part of the main assembly (but they weren't because their coverage wasn't high enough)?

Are sufficient details of methods and materials provided to allow replication by others?

Yes

Is the rationale for creating the dataset(s) clearly described?

Yes

Are the datasets clearly presented in a useable and accessible format?

Yes

Are the protocols appropriate and is the work technically sound?

Yes

Reviewer Expertise:

bioinformatics; insect genomics

I confirm that I have read this submission and believe that I have an appropriate level of expertise to confirm that it is of an acceptable scientific standard.
